# Unusual marine cyanobacteria/haptophyte symbiosis relies on N_2_ fixation even in N-rich environments

**DOI:** 10.1038/s41396-020-0691-6

**Published:** 2020-06-10

**Authors:** Matthew M. Mills, Kendra A. Turk-Kubo, Gert L. van Dijken, Britt A. Henke, Katie Harding, Samuel T. Wilson, Kevin R. Arrigo, Jonathan P. Zehr

**Affiliations:** 1 Earth System Science, Stanford University, Stanford, CA 94305 USA; 2 Ocean Science Department, University of California at Santa Cruz, Santa Cruz, CA 95064 USA; 3 Center for Microbial Oceanography: Research and Education, University of Hawaii at Manoa, Honolulu, HI 96822 USA

**Keywords:** Biogeochemistry, Microbial biooceanography

## Abstract

The microbial fixation of N_2_ is the largest source of biologically available nitrogen (N) to the oceans. However, it is the most energetically expensive N-acquisition process and is believed inhibited when less energetically expensive forms, like dissolved inorganic N (DIN), are available. Curiously, the cosmopolitan N_2_-fixing UCYN-A/haptophyte symbiosis grows in DIN-replete waters, but the sensitivity of their N_2_ fixation to DIN is unknown. We used stable isotope incubations, catalyzed reporter deposition fluorescence in-situ hybridization (CARD-FISH), and nanoscale secondary ion mass spectrometry (nanoSIMS), to investigate the N source used by the haptophyte host and sensitivity of UCYN-A N_2_ fixation in DIN-replete waters. We demonstrate that under our experimental conditions, the haptophyte hosts of two UCYN-A sublineages do not assimilate nitrate (NO_3_^−^) and meet little of their N demands via ammonium (NH_4_^+^) uptake. Instead the UCYN-A/haptophyte symbiosis relies on UCYN-A N_2_ fixation to supply large portions of the haptophyte’s N requirements, even under DIN-replete conditions. Furthermore, UCYN-A N_2_ fixation rates, and haptophyte host carbon fixation rates, were at times stimulated by NO_3_^−^ additions in N-limited waters suggesting a link between the activities of the bulk phytoplankton assemblage and the UCYN-A/haptophyte symbiosis. The results suggest N_2_ fixation may be an evolutionarily viable strategy for diazotroph–eukaryote symbioses, even in N-rich coastal or high latitude waters.

## Introduction

Primary production by marine phytoplankton is limited by N availability throughout much of the global open oceans [1]. As a result, phytoplankton have evolved metabolisms for utilizing different chemical forms of N (e.g., NO_3_^−^, ammonium (NH_4_^+^), or urea; [2]). One important N source for oligotrophic waters is N_2_ fixation, the conversion of N_2_ into biologically available ammonia, performed by some prokaryotes (diazotrophs), but no eukaryotes. Marine N_2_ fixation was once thought to be dominated by the tropical/subtropical cyanobacterium *Trichodesmium* sp. and cyanobacterial symbionts of some diatoms [3, 4]. This paradigm changed with the discovery that N_2_ fixation is also carried out by the unicellular N_2_-fixing cyanobacterial “group A” (UCYN-A; [5]), which lives in symbiosis with single-celled phytoplankton hosts related to the haptophyte *Braarudosphaera bigelowii* [6, 7]. UCYN-A is unusual in that it has a streamlined genome, lacking photosystem II, Rubisco, the Calvin Cycle, the TCA cycle, and NO_3_^−^ assimilation pathways [8, 9]. The haptophyte provides photosynthetically fixed C to UCYN-A in exchange for N supplied by UCYN-A from N_2_ fixation [7]. Two genetically distinct UCYN-A symbionts, UCYN-A1 and UCYN-A2, have similarly streamlined genomes [9], but are associated with morphologically and physiologically distinct haptophyte hosts [10, 11].

The fixation of N_2_ is energetically expensive, requiring large amounts of ATP and reductant compared with the assimilation of dissolved inorganic nitrogen (DIN) [12]. The sensitivity of marine N_2_ fixation to DIN concentrations is not well understood [13]. Culture-based studies show that N_2_ fixation by the cyanobacterial diazotroph *Trichodesmium* can be inhibited at elevated DIN concentrations (e.g., [14–16]), but notably growth and N_2_ fixation rates in the unicellular *Crocosphaera* can be insensitive to DIN availability [17–19]. The biogeography of the UCYN-A/haptophyte symbiosis extends into DIN-replete environments not typically considered important for N_2_ fixation, including cold high latitude waters [20, 21], coastal shelves [22, 23], and upwelling regions [24]. Recent evidence suggests that UCYN-A can grow in high NO_3_^−^ waters [25] and that N_2_ fixation in UCYN-A may not be completely inhibited by the presence of combined forms of DIN [26]. However, it is still not well understood whether growth of the UCYN-A/haptophyte symbiosis is supplemented by a N source other than UCYN-A N_2_ fixation when DIN is available to the haptophyte.

To determine the N source(s) used for growth by the UCYN-A1/ and UCYN-A2/haptophyte symbioses, we conducted a series of experiments in the southern coastal waters of the California Current System (CCS; Table 1). A fully replicated design (details below) was implemented to assess the effects of NO_3_^−^ or NH_4_^+^ additions on bulk community responses (chlorophyll *a* (Chl *a*) and particulate organic carbon (POC) and nitrogen (PON) concentrations), as well as N_2_ fixation, C fixation, and DIN uptake rates by the bulk phytoplankton assemblage and the UCYN-A/haptophyte symbioses specifically. The stable isotope tracers ^15^N_2_, ^15^NO_3_^−^, ^15^NH_4_^+^ and H^13^CO_3_^−^ were used to measure N_2_ fixation, DIN uptake, and C fixation rates, respectively, by the phytoplankton assemblage. The cell-specific UCYN-A/haptophyte symbioses were measured using sublineage-specific catalyzed reporter deposition fluorescence in-situ hybridization (CARD-FISH) assays [27, 28] combined with nanoscale secondary ion mass spectrometry (nanoSIMS). The experiments were designed to investigate whether UCYN-A continues to fix N_2_ when NO_3_^−^ and NH_4_^+^ are readily available, if the haptophyte host takes up NO_3_^−^ and NH_4_^+^, and if responses to NO_3_^−^/NH_4_^+^ additions were UCYN-A/haptophyte sublineage-specific.

**Table 1:** Summary of T_0_ parameters for NO_3_^−^ and NH_4_^+^ addition experiments and T_48_ treatment concentrations.

Experiment	Lat, Lon (ddm)	Date	NO_3_^−^+ NO_2_^−^ (µM)	NO_2_^−^(µM)	NH_4_^+^ (µM)	PO_4_^3−^ (µM)	Si (µM)	T (°C)	Treatment T_48_ NO_3_^−^ + NO_2_^−^ (µM)	Treatment T_48_ NH_4_^+^ (µM)
NO3.1	32.84, −117.531	May 3–5, 2017	0	nm	0.29	nm	16.7 ± 0.16	0.14 ± 0.01	0.8	nm
NO3.2	28.289, −115.914	Oct 6–8, 2017	nm	nm	0.14	0.17	21.5 ± 0.00	0.07 ± 0.01	1.45 ± 0.03	nm
NO3.3	30.358, −116.359	Oct 7–9, 2017	nm	nm	0.13	3.18	19.3 ± 0.22	0.16 ± 0.02	1.42 ± 0.05	nm
NH4.1	32.867, −117.256	May 10–12, 2018	0.13^a^	0.25^a^	0.29^a^	9.02^a^	14.9 ± 0.14^a^	3.94 ± 0.42	nm	0.80 ± 0.43

NO3.1 was conducted on the R/V Robert Gordon Sproul in the proximity of the SIO pier in May 2017. NO3.2 and NO3.3 were conducted at stations off the coast of Baja California Sur, Mexico in October 2017. NH4.1 was conducted at the SIO pier in May 2018 (Fig. S1).

*nm* not measured.

^a^From SCOOS monitoring data at SIO pier on May 10, 2018.

## Materials and methods

### DIN experiments: experimental design and sampling

Four experimental manipulations were conducted during 2017 and 2018 in the southern coastal waters of the CCS; three experiments (NO3.1, NO3.2, NO3.3) were NO_3_^−^ addition experiments and one was an NH_4_^+^ addition experiment (NH4.1). NO3.1–3 were conducted at three different stations aboard the R/V Gordon Sproul during two research cruises in 2017 that transited off the coast of Southern California and Baja California Sur, Mexico, while NH4.1 was conducted on the Scripps Institute of Oceanography pier (Table 1, Fig. S1).

For NO3.1–3, surface water was pumped into 40 L carboys, housed in an on-deck laboratory container, using a pneumatic (PVDF and Teflon) diaphragm pump (Wilden Pump and Engineering, Grand Terrace, CA), to allow mixing of the seawater before being randomly dispensed into acid-cleaned 4 L polycarbonate bottles (Thermo Scientific™ Nalgene™, Waltham, MA). Grazers were removed using 150 µm Nitex™ plankton netting (BioQuip, Rancho Dominguez, CA). The bottles were then incubated in triplicate with or without an addition of NO_3_^−^ (2 µmol L^−1^ final concentration) at T_0_, according to the experimental design in Fig. S2. Incubation bottles were placed in a flow-through surface seawater incubator, amended with neutral density screening to attenuate incident light to 20% of the surface irradiance. Incubations lasted 48 h, with initial rate measurements between 0 and 24 h and final rate measurements between 24 and 48 h. Final concentrations of ^15^N- and ^13^C-labeled substrates for rate measurements are detailed in Table S2. At each time point, bottles were sacrificed and subsampled for measuring Chl *a* concentration, dissolved and particulate nutrient concentrations, bulk CO_2_ and N_2_ fixation rates, inorganic N uptake rates, flow cytometry, diazotroph abundance (qPCR-based estimates using assays targeting the *nifH* gene), and UCYN-A/haptophyte symbiosis cell-specific N_2_ fixation, CO_2_ fixation and NO_3_^−^ uptake rates (CARD-FISH, nanoSIMS). Unlabeled initial samples were used to determine the atom% ^15^N- and ^13^C-normal of the unenriched bulk community and UCYN-A/haptophyte symbioses.

For NH4.1, surface water was pumped into 40 L carboys from the waters surrounding the SIO Pier using a pneumatic (PVFD and Teflon) diaphragm pump (Wilden Pump and Engineering), then randomly dispensed into acid-cleaned 2 L polycarbonate bottles (Thermo Scientific™ Nalgene™). Grazers were removed using 150 µm Nitex™ plankton netting (BioQuip, Rancho Dominguez, CA). The bottles were then incubated with or without an NH_4_^+^ addition (2 µmol L^−1^ final concentration) at T_0_, according to the experimental design in Fig. S3. Incubation bottles were placed in a flow-through surface seawater incubator, amended with neutral screening to attenuate incident light to 20% of the surface irradiance. Incubations lasted 48 h, with N_2_ fixation initial rate measurements between 0 and 24 h and final rate measurements between 24 and 48 h. For NH_4_^+^ uptake rates, initial rates were measured between 0 and 6 h, and final rates in NH_4_^+^-treatments were measured between 45 and 51 h. Incubation times (6 h) were chosen to ensure detection of isotope enrichments while minimizing isotope dilution, as recommended in Glibert [29]. Final concentrations of ^15^N-labeled substrates for rate measurements are detailed in Table S2. At each time point, bottles were sacrificed and subsampled for Chl *a* concentration, dissolved and particulate nutrient concentrations, bulk CO_2_ and N_2_ fixation rates, inorganic N uptake rates, diazotroph abundance (DNA), and UCYN-A/haptophyte symbiosis cell-specific N_2_ fixation, CO_2_ fixation, and NO_3_^−^ uptake rates (CARD-FISH, nanoSIMS). Unlabeled initial samples were used to determine the atom% ^15^N- and ^13^C-normal of the unenriched bulk community and UCYN-A/haptophyte symbioses.

### Dissolved and particulate nutrient analyses

Samples for the measurement of NO_3_^−^ + NO_2_^−^, PO_4_^3-^, and Si(OH)_4_ concentrations were filtered through precombusted (450 °C for 4.5 h) 25 mm GF/F filters and stored in acid-cleaned Falcon^TM^ tubes (Thermo Fisher Scientific) at −20 °C until analysis using standard techniques [30] on a Lachat QuikChem 8000 Flow Injection Analyzer. Samples for the analysis of POC and PON were filtered onto precombusted (4 h @ 450 °C) 25 mm Whatman GF/F filters. Blank filters were made by filtering ca. 25 ml filtered (0.2 µm) seawater and were processed the same as the particulate samples. The filters were dried (60 °C) and stored at room temperature until analysis. Prior to analysis the samples were fumed with concentrated HCl, dried at 60 °C for 24 h, packed into tin capsules (Costech Analytical Technologies Inc. Valencia, CA) and analyzed on an Elemental Combustion System (Costech Analytical Technologies) interfaced to a Thermo Finnigan Delta V Advantage isotope ratio mass spectrometer (Thermo Fisher Scientific) at the SOEST Biogeochemical Stable Isotope Facility at the University of Hawai’i, Manoa. Fluorometric analysis of Chl *a* was measured [31] using a Turner Fluorometer TD-700 (Turner Designs, Inc., San Jose, CA).

### ^15^N_2_ fixation and C fixation rate measurements

We measured ^15^N_2_ incorporation into biomass using a “dissolution approach” amended from Mohr et al. [32] and Wilson et al. [33]. ^15^N_2_-enriched seawater was generated in batches for each experiment by filtering seawater collected from the experimental site through a Pall 0.2 μm Acropak 1550 Capsule Filter with Supor Membrane (Pall Corp, Port Washington, New York). The filtered seawater (FSW) was degassed under vacuum for 30–60 min, while being stirred. Degassed water was quickly transferred via siphon into 2 or 4 L polycarbonate bottles and capped with PTFE-lined (Ace Glass Incorporated, Vineland, NJ) septa and caps. Bottles were then overpressurized by injecting between 20 and 30 mL of ^15^N_2_ gas (Cambridge Isotope Laboratories, Tewksbury, MA) and agitated at room temperature on a rocking plate (NH4.1) or by the motion of the ship (NO3.1–NO3.2) for >12 h. To verify the atom% enrichment of each batch of ^15^N_2_ tracer-labeled seawater, duplicate 12 ml Exetainers® (Labco, Lampeter, Ceredigion, U.K) were filled immediately prior to the initiation of each experimental incubation for subsequent membrane inlet mass spectrometer (MIMS) analysis at the University of Hawai'i at Manoa according to Wilson et al. [33]. The quantity of nitrogen isotopes (i*.*e., N masses equivalent to 28, 29, and 30) was measured in each batch of ^15^N_2_ enriched seawater. Calibration of the MIMS was achieved by the analysis of a 1 L reservoir of air-equilibrated filtered (0.2 µm) seawater with a known salinity and a temperature of 23 °C [34]. The final atom% enrichment in the seawater incubations averaged 5.9 ± 1.7 (with a total range of 2.4–8.6 atom% enrichment).

Incubation bottles received 400 mL (NO3.1–NO3.3) or 200 mL (NH4.1) of ^15^N_2_-enriched FSW to initiate the experiment. Each incubation bottle also received NaH^13^CO_3_ (Cambridge isotopes) according to Table S2. Following a 24 h incubation period, samples were gently vacuum filtered onto a combusted 25 mm glass fiber filter and stored at −20 °C until preparation for analysis. Samples were dried, acidified, and prepared for analysis as the POC/PON samples above. The ^15^N_2_ and ^13^CO_2_ enrichment of the particulate material was measured using an Elemental Combustion System CHNS-O (ECS 4010) (Costech Analytical Technologies, Inc. Valencia, CA) interfaced to a Thermo Scientific Delta V Advantage isotope ratio mass spectrometer at the SOEST Biogeochemical Stable Isotope Facility at the University of Hawai'i at Manoa. LOD, estimates of error and minimum quantifiable rates (MQR) were calculated as in Gradoville et al. [35], and are detailed in Tables S13 and S14.

### ^15^NO_3_^−^ and ^15^NH_4_^+^ uptake bulk community rate measurements

^15^N incorporation into biomass from DIN substrates (NO_3_^−^ and NH_4_^+^) was measured in both control and treatment incubations. ^15^N substrate additions (^15^NO_3_^−^ or ^15^NH_4_^+^; Cambridge Isotope Laboratories) were made with the goal of enriching the ambient pool ~4–10% (Table S2). For the NO_3_^−^-addition experiments, ambient NO_3_^−^ concentrations were estimated using NO_3_^−^-temperature relationships for California Current waters [36], while for NH4.1, ambient NH_4_^+^ concentrations were estimated based on historical data from the Southern California Coastal Ocean Observing System (SCCOOS). Incubation bottles receiving ^15^NO_3_^−^ or ^15^NH_4_^+^ also received NaH^13^CO_3_ (Cambridge Isotope Laboratories) according to Table S2. Following a 24 h incubation period for NO3.1–NO3.3 and a 6 h incubation period for NH4.1, samples were gently vacuum filtered onto a precombusted 25 mm glass fiber filter and stored at −20 °C until preparation and analysis as described above for the N_2_ fixation measurements.

Actual ambient concentrations were typically lower than estimates made using this approach. In cases where ambient NO_3_^−^ concentrations were below detection limits (LOD = 0.1 µmol L^−1^), substrate pool enrichments were calculated using the LOD [29], and should be considered maximum uptake rates. Ambient NH_4_^+^ concentrations were measured as part of the SCCOOS monitoring program and isotope enrichments were calculated from these measurements.

Potential isotope dilution effects that may result from NH_4_^+^ regeneration during NH4.1 were calculated using regeneration rates measured in Southern California Bight waters by Bronk and Ward [37] (Table S3). Dilution of the NO_3_^−^ isotope pool was not likely to be significant as surface water rates of nitrification in Southern California current waters are typically very low [37].

**Fig. 1:**
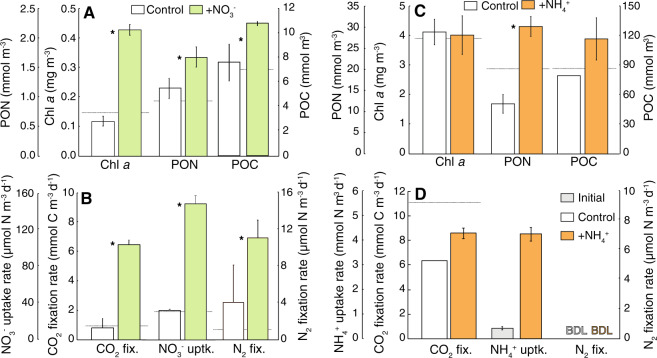
Bulk particulate^,^ and biological rate responses to DIN additions in Southern California Coastal Current Waters. **a**, **b** Data from NO3.1 conducted in May 2017. **c**, **d** Data from NH4.1 conducted in May 2018. **a** PON, Chl *a* and POC concentrations in control and in 2 µM +NO_3_^−^-treatments. **b** Whole community NO_3_^−^ uptake rate, CO_2_ and N_2_ fixation rates in control and in 2 µM +NO_3_^−^-treatments. **c** PON, Chl *a* and POC concentrations in control and in 2 µM NH_4_^+^-treatments. **d** Whole community NH_4_^+^ uptake rate, CO_2_ and N_2_ fixation rates in control and in 2 µM +NH_4_^+^-treatments. All error bars are standard deviations from biological triplicates. Initial values are denoted with dotted gray lines. BDL indicates measurements below detection and asterisk symbol indicates treatment means are significantly different (*p* < 0.05, α = 0.05). Note all bars are time final control and treatment responses, except in (**c**) where the control NH_4_^+^ uptake rate sample was lost and the initial is shown as a gray bar.

Phytoplankton uptake of NO_3_^−^ and NH_4_^+^ are associated with strong isotopic fractionation effects that can lead to the accumulation of an isotopically heavy DIN pool [38]. Assimilation of this isotopically heavy DIN during a ^15^N_2_ fixation incubation leads to an overestimation of N_2_ fixation rates, or even a false positive for N_2_ fixation. This effect is likely insignificant in oligotrophic waters, but not in nutrient rich waters that are transiently poor in nutrients due to phytoplankton consumption. These were the conditions during NH4.1, where concentrations of NO_3_^−^ were 0.46 µmol L^−1^ at the time of the experiment but were 6 µmol L^−1^ 10 days prior to beginning the experiment. However, we detected no ^15^N enrichment of the PON pool in the ^15^N_2_ incubations and were thus unable to calculate bulk N_2_ fixation rates (Fig. 1d, Table S4). As such we do not consider isotopic fractionation of the DIN pool a concern in this experiment. All other experiments were conducted under nutrient limited conditions, thus ambient DIN pools were unlikely enriched due to isotope fractionation.

### Measuring UCYN-A/haptophyte symbioses single-cell rates

Experiments with UCYN-A/haptophyte symbioses present at suitable abundance for nanoSIMS analyses were first identified using qPCR targeting the UCYN-A1 and UCYN-A2 *nifH* gene (Supplemental text, Table S5). All UCYN-A/haptophyte symbioses single-cell rates were measured using CARD-FISH to visualize and target the UCYN-A/haptophyte symbioses coupled to nanoSIMS to measure the incorporation of ^15^N or ^13^C into individual associations. Subsamples (95 mL) from each incubation bottle were fixed with 5 mL of sterile filtered 37% formaldehyde (MilliporeSigma), fixed for between 1 and 48 h at 4 °C in the dark, and then filtered under low vacuum onto a 0.6 µm polycarbonate filter (MilliporeSigma). Filters were air dried and frozen at −80 °C until processing.

#### CARD-FISH

Fluorophore-containing tyramides were deposited into host and symbiont cells, using 5′-horseradish peroxidase (HRP)-labeled oligonucleotide probes (Biomers.net, Inc., Ulm/Donau, Germany) targeting each UCYN-A/haptophyte sublineage, in combination with helper and competitor probes for both symbionts and hosts (Biomers.net), as described in [28] and Table S1. Briefly, cells were attached to filters with 0.1% Ultrapure agarose (Life Technologies, Carlsbad, CA), then permeabilized in a two-step process with lysozyme achromopeptoidase (MilliporeSigma) solution. Hybridizations with HRP-labeled probes were carried out in hybridization buffer at 46 °C for the host hybridizations and 35 °C for the symbiont hybridizations. Unincorporated probe was removed with several wash steps with a buffer preheated to 2 °C greater than the hybridization temperature. The tyramide signal amplification (TSA) step deposited fluorophore-containing tyramides in the presence of an amplification buffer and hydrogen peroxide. The haptopyte host was labeled with the Alexa 488 fluorophore (Biomers.net), and the symbiont was labeled with the Cy3 fluorophore (Biomers.net). Post amplification, filters were washed with PBS, hydrogen peroxide was deactivated with 0.01 M HCl, then filters were rinsed with Milli-Q™ (MilliporeSigma) water. After the second round of hybridization, TSA, and washing, filters were dried, and counterstained with ProLongTM Diamond Antifade Mountant with DAPI (Molecular Probes, Eugene, OR).

Filters were visualized on a Zeiss Axioplan epifluorescence microscope (Oberkochen, Germany) equipped with digital imaging to verify that both host and symbiont hybridizations were optimal, allowing for positive identification and mapping of active (vital) UCYN-A/haptophyte symbioses. The filters containing the successfully hybridized cells were then gently rinsed with milli-Q water and then placed cell side down onto an alphanumeric labeled gridded silicon wafer (1.2 × 1.2 cm with a 1 × 1 mm raster, Pelcotec^TM^ SFG12 Finder Grid Substrate, Ted Pella, Redding, CA). The wafer was then placed into a −80 °C freezer for 5–10 min before being removed and the filter peeled off. Particulate matter remaining on the wafer was then allowed to air dry before multiple UCYN-A/haptophyte targets were randomly imaged and mapped at 40x using the above-mentioned epifluorescence microscope.

#### NanoSIMS analysis and rate calculations

The maps produced from the CARD-FISH imaging were used to locate the UCYN-A/haptophyte targets on a Cameca nanoSIMS 50 L at the Stanford Nano Shared Facilities (Stanford, CA) using the CCD camera. Symbioses selected for nanoSIMS analysis were randomly selected from the mapped cells for analysis based on ease of localization on the silicon wafer, clarity of the secondary electron image, and magnitude of the ^12^C^14^N^−^ signal (i.e., if the image was difficult to focus or if there was sample charging that obscured the signal, a different cell was selected) (Fig. S6). Image fields were then rastered with a 16 keV Cesium primary ion beam (~5 pA). Primary ions were focused into ~120 nm spot diameter and all measurements were made at a mass resolving power of approximately 8000. We rastered an area with 256 × 256 pixels over the chosen raster size with a dwell time of 1 ms per pixel. We collected images of ^12^C^−^, ^13^C^−^, ^12^C^14^N^−^ and ^12^C^15^N^−^ over 30–100 planes. Both UCYN-A and haptophytes were selected as regions of interest (ROI) using the image analysis software Look@nanoSIMS [39]. Isotope ratios of UCYN-A and the hosts were calculated as the ratio of the sum of total ion counts within the ROIs for each pixel over all recorded planes of the enriched and unenriched isotopes (i.e., ^13^C^−^/^12^C^−^ and ^12^C^15^N^−^/^12^C^14^N^−^). Corrections for beam and stage drift were made for all scans. Rates were determined as follows:

$$\rho \left( {fmol\,cell^{ - 1}d^{ - 1}} \right) = \frac{{At\% _{sample} - At\% _{normal}}}{{\left( {At\% _{substrate} - At\% _{normal}} \right) \times T}} \times B,$$

Where *ρ* equals the absolute uptake rate per cell, *At%*_*sample*_, *At%*_*normal*_, and *At%*_*substrate*_ equal the atom% ^15^N or ^13^C of the enriched (T_48_) or unenriched (T_0_) sample and the respective added ^15^N or ^13^C enriched substrate. Substrate enrichments were measured for N_2_ following Kana et al. [40] and calculated for DIN and HCO_3_^−^ based on ambient concentrations. In addition, *T* is time in days and *B* is the per cell biomass estimates determined from biovolumes as in Krupke et al. [26] and converted to units of N using C:N estimates from Martinez-Perez et al. [11]. Detection limits, estimates of error and MQR were calculated as in Montoya et al. [41] and Gradoville et al. [35] (Tables S13 and S14). N_2_ rates, C fixation rates, and NO_3_^−^/NH_4_^+^ uptake rates for both the symbiont and hosts were calculated individually and then summed to get total symbiosis rate for either the symbiont (N_2_ fixation) or host (C fixation, NO_3_^−^/NH_4_^+^ uptake). Measuring the isotopic abundance of the symbionts and hosts individually allowed for the inclusion of N transferred from the UCYN-A to the haptophyte and C transferred from the host to the symbiont. Care was taken to measure samples from the same experiments within the same measurement period so as to minimize machine variance between measurement periods.

### Statistical analyses

Single factor analysis of variance (ANOVA) was used to evaluate the significance of treatment effects (Control, +NO_3_^−^, + NH_4_^+^) on Chl *a* (Table S6), POC (Table S7), PON (Table S8), *nifH*-based UCYN-A abundance (Table S9), and whole community N_2_ (Table S10), and C fixation (Table S11), and NO_3_^−^ uptake rates (Table S12). Further ANOVA analyses tested the impact of the NO_3_^−^ and NH_4_^+^ additions on the single-cell N_2_ and C fixation rates in each experiment (Tables S15–22). Treatment responses were considered significantly different at the α = 0.05 significance level.

## Results

The phytoplankton assemblage response to the addition of NO_3_^−^ indicated they were N-limited throughout the study region. Bulk responses to NO_3_^−^ additions included a 1.7–4.5-fold stimulation of Chl *a* and a 1.3 ± 0.2 and 1.5 ± 0.4-fold increase in POC and PON concentrations, respectively (Figs. 1a, S4A, B). In addition, bulk C fixation rates, maximum NO_3_^−^ uptake rates, and N_2_ fixation rates (in NO3.1) increased up to 13-fold, 9.5-fold, and 4-fold (Figs. 1b, S4C, D), respectively. The diazotroph assemblage at T_0_ in NO3.1 included UCYN-A1, UCYN-A2, *Richelia* associated with the diatom *Hemiaulus* and a putative γ-proteobacterial diazotroph, gamma A (Table S23), each of which may have contributed to bulk N_2_ fixation rates. UCYN-A1 abundance was higher in +NO_3_^−^ treatments than controls at both time points, despite being lower than T_0_ abundances (Table S5).

**Fig. 2:**
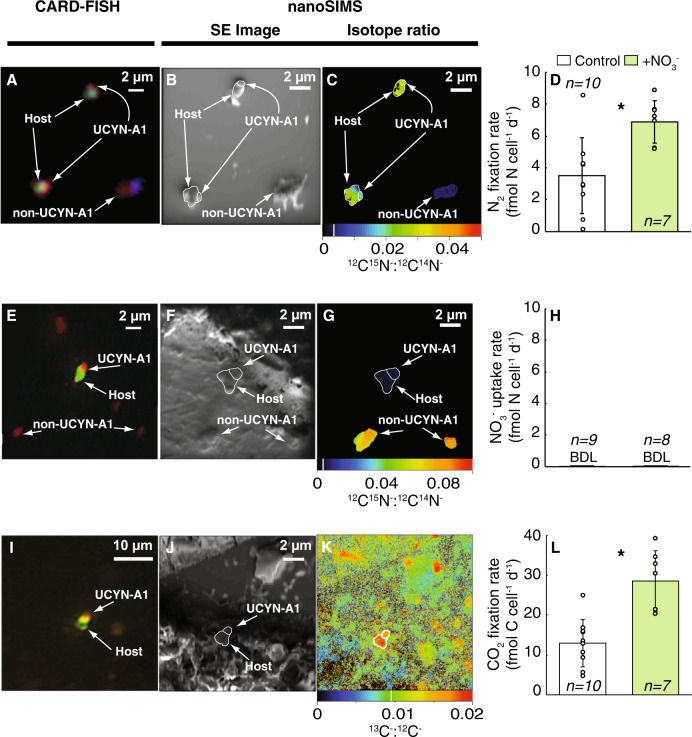
Individual UCYN-A1/haptophyte cells had higher rates of N_2_ and C fixation, but no NO_3_^−^ assimilation. Data from NO3.1; see Fig. S2 for NO3.2 and NO3.3. **a**–**c** Example images of cells from NO_3_^−^ treatments, where ^15^N enrichment from ^15^N_2_ fixation was measured. **d** Cell-specific rates of N_2_ fixation in control and NO_3_^−^ treatments. **e**–**g** Example images of cells from NO_3_^−^ treatments, where ^15^N enrichment from ^15^NO_3_^−^ uptake was measured. **h** Cell-specific rates of NO_3_^−^ uptake in control and NO_3_^−^ treatments. **i**–**k** Example images of cells from NO_3_^−^ treatments, where ^13^C enrichment from H^13^CO_3_^−^ was measured. **l** Cell-specific rates of CO_2_ fixation in control and NO_3_^−^ treatments. **a**, **e**, **i** Epifluorescence micrographs of UCYN-A1 (red) and the haptophyte host (green) stained with CARD-FISH probes [28] and DAPI (blue). **b**, **f**, **j** Corresponding secondary electron (SE) images displaying target cells. Isotope ratio images acquired from nanoSIMS analysis showing isotopic enrichment in ^15^N from a ^15^N_2_ incubation (**c**), ^15^N from a ^15^NO_3_^−^ incubation (**g**), and ^13^C from a H^13^CO_3_^-^ incubation (**k**) incubated samples. Data from individual cells (circles) are included in (**d**) and (**l**). Note the difference in scales. Non-UCYN-A1/haptophyte cells are identified for contrast in (**c**) and (**g**). Natural abundances are noted with a white line on the color scale bar in (**c**), (**g**) and (**k**). Rates represent integration over a diel cycle (final 24–48 h). BDL indicates measurements below detection and asterisk symbol indicates treatment means are significantly different (*p* < 0.05, α = 0.05).

Surprisingly, under these experimental conditions, the haptophyte host of UCYN-A1 did not assimilate NO_3_^−^ (Figs. 2e–h, S5D). The host, however, did exhibit significantly higher C fixation rates in the NO_3_^−^ treatment in NO3.1 (*p* < 0.01; Fig. 2i–l, Table S19) indicating that haptophyte C fixation was indirectly stimulated by the NO_3_^−^ addition. In addition, the average per cell rate of N_2_ fixation in the NO_3_^−^ treatment relative to the control in NO3.1 (*p* < 0.01, Fig. 2a–d, Table S15). In general, average N_2_ and C fixation per cell rates were higher in NO_3_^−^ treatments, but not always statistically significant (Tables S16 and S20). Notably, the haptophyte host of UCYN-A2 also did not assimilate NO_3_^−^ (Fig. S5D, Table S12).

**Fig. 3:**
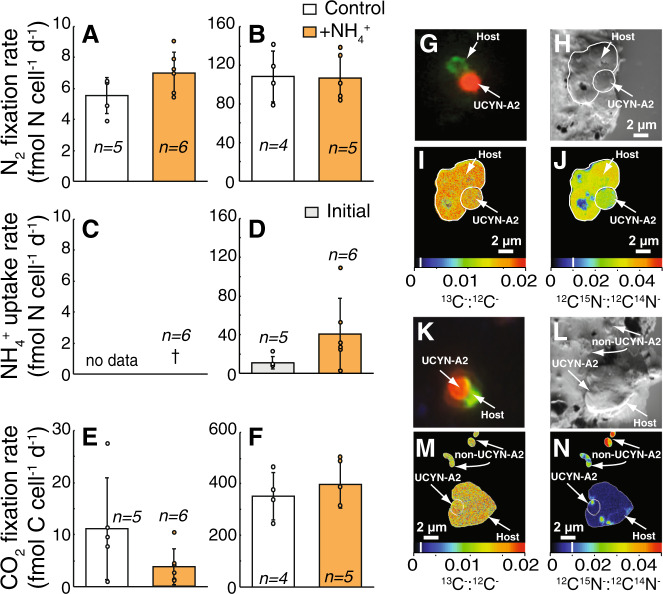
N_2_ fixation in UCYN-A1/ and UCYN-A2/haptophyte symbioses is not inhibited in the presence of NH_4_^+^. Cell-specific N_2_ fixation rates for the UCYN-A1/haptophyte symbiosis (**a**) and UCYN-A2/haptophyte symbiosis (**b**) in control and +NH_4_^+^-treatments from NH4.1. Cell-specific NH_4_^+^ uptake rates for the UCYN-A1/haptophyte symbiosis (**c**) and UCYN-A2/haptophyte symbiosis (**d**) in control and +NH_4_^+^-treatments. Cell-specific CO_2_ fixation rates for the UCYN-A1/haptophyte symbiosis (**e**) and UCYN-A2/haptophyte symbiosis (**f**) in control and +NH_4_^+^-treatments. **g**–**j** Example images of a UCYN-A2/haptophyte symbiosis from NH_4_^+^-treatments, where ^15^N enrichment from ^15^N_2_ fixation was measured. **k**–**n** Example images of a UCYN-A2/haptophyte symbiosis from NH_4_^+^-treatments, where ^15^N enrichment from ^15^NH_4_^+^ uptake was measured. **g**, **k** Epifluorescence micrographs of UCYN-A2 (red) and the haptophyte host (green) stained with CARD-FISH probes [28] and DAPI (blue). **h**, **l** Corresponding secondary electron (SE) images displaying target cells. Isotope ratio images acquired from nanoSIMS analysis showing isotopic enrichment in ^13^C from a H^13^CO_3_^−^ incubation (**i**, **m**), ^15^N from a ^15^N_2_ incubation (**j**), and ^15^N from a ^15^NH_4_^+^ incubation (**n**). Non-UCYN-A2/haptophyte cells are identified for contrast in (**l**–**m**). Data from individual cells (circles) are included in (**a**–**f**). N_2_ fixation rates represent integration over a diel cycle (final 24–48 h); while NH_4_^+^ uptake rates were measured over a 6-h period. Note treatment responses are compared to initials in (**d**). The Dagger symbol indicates below minimum quantifiable rate. Natural abundances are noted with a white line on the color scale bar in (**i**), (**j**), (**m**), and (**n**).

Since the symbiosis did not assimilate NO_3_^−^, we also tested for the uptake of NH_4_^+^, the most reduced and typically preferred N substrate for phytoplankton [2]. NH_4_^+^-addition experiments were conducted in coastal CCS waters (Figs. S1, S3) during the late stages of a bloom of the dinoflagellate *Lingulodinium polyedra*, with relatively high initial DIN concentrations (0.46 µmol L^−1^ NO_3_^−^ + NO_2_^−^  and 0.25 µmol L^−1^ NH_4_^+^) and relatively abundant UCYN-A/haptophyte populations (10^4^–10^5^ *nifH* copies L^−1^; Table S5). Chl *a* concentrations and bulk C fixation rates were not stimulated by NH_4_^+^ additions (Fig. 1c, d), but POC/PON concentrations and NH_4_^+^ uptake rates increased 1.5–2.5- and 5.5-fold, respectively (Fig. 1c, d, Tables S6, S8, S12, Supplemental text). Despite undetectable bulk rates of N_2_ fixation (Fig. 1d; Table S4), per cell rates of N_2_ and C fixation were measured in both UCYN-A1/ and UCYN-A2/haptophyte symbioses, with no difference in rates between controls and NH_4_^+^ additions (Fig. 3a, b, e, f, g–j, Tables S13 and S14, Tables S17 and S18, Tables S21 and 22). Surprisingly, NH_4_^+^ uptake rates were low relative to the N_2_ fixation rates in both UCYN-A hosts (Fig. 3c, d, k-n, Table S13), even when accounting for possible isotope dilution effects due to NH_4_^+^ regeneration (see Methods and Table S3). UCYN-A1 host NH_4_^+^ uptake rates were not quantifiable (Fig. 3c) while UCYN-A2 host NH_4_^+^ uptake rates were ~3–10-fold less than measured N_2_ fixation rates (Fig. 3d).

## Discussion

Our findings indicate that the net benefits of maintaining N_2_ fixation must outweigh the costs when compared with the assimilation of DIN for this symbiotic association. N_2_ fixation in the UCYN-A/haptophyte symbioses provides N to the host cell under both N-limited and N-replete conditions, contrary to other marine diazotrophs (e.g., *Trichodesmium*, *Crocosphaera*, and the heterocyst-forming symbiont *Richelia* associated with *Rhizosolenia*) for which NO_3_^−^ and NH_4_^+^ utilization can meet significant proportions of their N requirements when available [14, 15, 17, 18, 42].

The lack of NO_3_^−^ assimilation in a eukaryotic alga is highly unusual. In addition, the low rates of NH_4_^+^ assimilation by the haptophyte host are also unusual, but could result from high intracellular NH_3_ concentrations, due to UCYN-A N_2_ fixation, which may create a gradient that prevents uptake [43]. Marine phytoplankton typically possess the metabolic capabilities to assimilate NO_3_^−^, although uptake rates and internal storage capabilities vary between species [44]. NO_3_^−^ assimilation is common in other haptophyte lineages, including *Emiliania huxleyi* [45] and *Prymnesium parvum* [46]; however nothing is known about the N utilization strategies in *B. bigelowii* beyond N acquisition from the symbiont [7]. There are some examples of algae that do not appear to assimilate NO_3_^−^. Although not closely related to *B. bigelowii*, *Chrysochromulina breviturrita*, a freshwater haptophyte, cannot grow on NO_3_^−^ as its sole N source, and is assumed to have a specialized N metabolism due to the acidic conditions where it lives [47]. The only other marine eukaryotic alga reported not to assimilate NO_3_^−^ are mixotrophs from the family *Ochromonadaceae*, which acquire most of their required N by consuming prey and have potentially lost the genetic capability for NO_3_^−^ assimilation and urea transport [48, 49].

It cannot be determined whether the lack of NO_3_^−^ uptake results from genomic streamlining or metabolic control until genomes and/or transcriptomes from host cells are obtained. However, the observation that both haptophyte hosts do not assimilate NO_3_^−^ suggests that the haptophyte’s last common ancestor may not have relied on the assimilation of NO_3_^−^ to meet their N demands prior to divergence [28]. Thus, N-acquisition strategies may be important in either establishing or maintaining symbioses between diazotrophs and eukaryotes, especially in the oligotrophic marine environment.

**Fig. 4:**
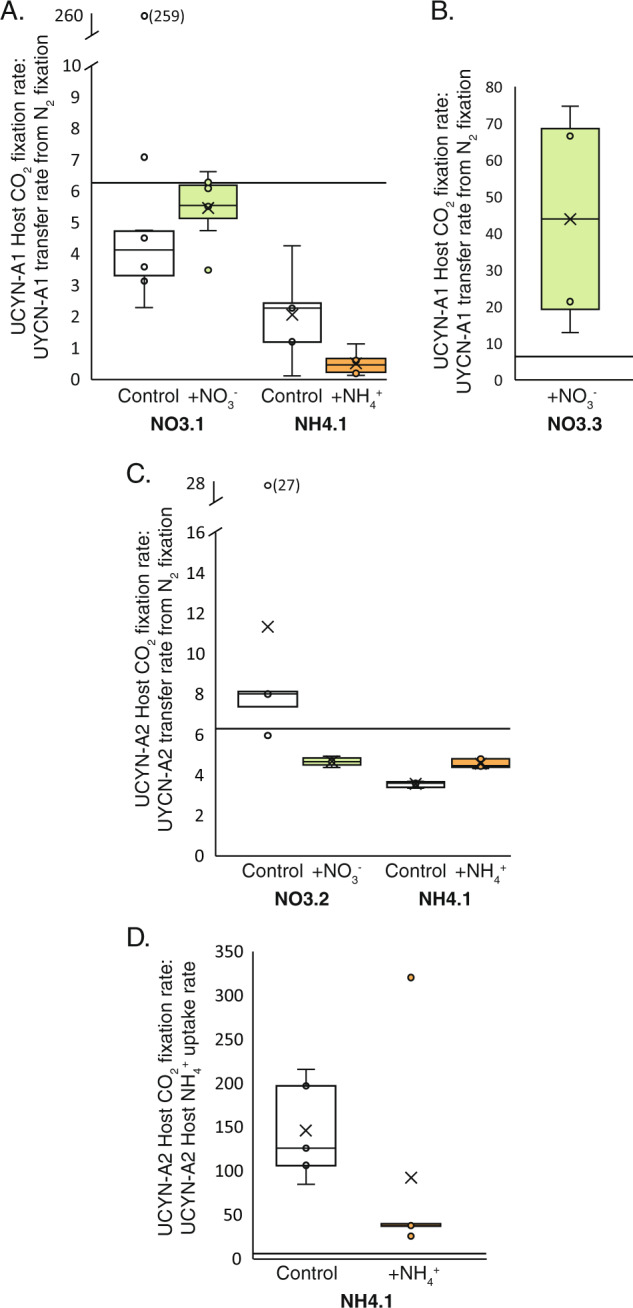
N_2_ fixation by UCYN-A supports the haptophyte host’s N demands and cellular growth in DIN-deplete and DIN-replete waters. **a** Ratio of host CO_2_ fixation rate to N transfer rate from N_2_ fixation in the UCYN-A1/haptophyte symbiosis in NO3.1 and NH4.1. Note the break in *y*-axis. Mean value (30) for NO3.1 control falls in break. **b** Ratio of host CO_2_ fixation rate to N transfer rate from N_2_ fixation in the UCYN-A1/haptophyte symbiosis in NO3.3 NO_3_^−^ treatment. **c** Ratio of host CO_2_ fixation rate to N transfer rate from N_2_ fixation in the UCYN-A2/haptophyte symbiosis in NO3.2 and NH4.1. **d** Ratio of host CO_2_ fixation rate to NH_4_^+^ uptake rate ratio in the UCYN-A2/haptophyte in NH4.1. Cellular C:N ratio of 6.3 (solid line; [11]) is plotted on all graphs. Values that fall near or under the 6.3 ratio line indicate that cellular growth can be met by N_2_ fixation. Data points that fall above the 6.3 ratio line indicate that cellular growth cannot be met by the N source.

These experiments demonstrate that UCYN-A N_2_ fixation supplies the needed N to support host cellular demands in both N-deplete and N-replete conditions. This is evident when comparing the C fixation rate to N transfer rate ratio (i.e., the ratio of the C fixation rate to the rate that N from UCYN-A N_2_ fixation was transferred to the host cell) to the best estimate of the UCYN-A/host symbiosis cellular C:N (6.3; [11]). In almost all instances the C fixation rate to N transfer rate ratio (Fig. 4a, c) was less than the cellular ratio, indicating that N_2_ fixation met host N demands. In contrast, C fixation rates in UCYN-A2 were 32–75-fold greater than host NH_4_^+^ uptake rates (Fig. 4d) indicating that NH_4_^+^ uptake cannot solely meet haptophyte N demands. Thus, even in N-replete waters, N_2_ fixation supported the UCYN-A1 and UCYN-A2 host requirements and NH_4_^+^ uptake was a minor source of N for the symbiosis, despite it being energetically preferable [50, 51].

Notably, UCYN-A1 N_2_ fixation could not fulfill the N required by the UCYN-A1 haptophyte host in NO3.3 (C fixation rate to N transfer rate ratio greater than 6.3; Fig. 4b), suggesting the symbiosis requires exogenous N sources under some conditions. Potential N sources include dissolved organic N (DON) or acquiring N through mixotrophy. DON utilization by *E. huxleyi* has been demonstrated to be an important source of N in nutrient-depleted surface ocean waters [52, 53]. Phagotrophy may be unusual in some haptophyte lineages [54, 55]; however, haptophytes have also been identified as important grazers in coastal systems [56, 57]. UCYN-A haptophyte hosts have been identified as active predators of *Prochlorococcus* and *Synechococcus* in the North Pacific by Frias‐Lopez et al. [58], although nothing was known about the symbiosis or the 18S rRNA gene sequences of the hosts at that time, so they were originally classified as unknown *Prymnesiophycaea*.

There are very few single-cell measurements of C and N transfer rates in the UCYN-A/haptophyte symbiosis, and those presented here are the first from associations living in coastally-influenced waters. Thus, we do not have a good understanding of the range or variability of these rates. Our rates are an order of magnitude greater than those reported by Krupke et al. [26]; however they used a C:N estimate for the UCYN-A/haptophyte symbiosis of 8.6, vs. while we applied a C:N of 6.3 as measured by [11] for our calculations. A higher C:N results in a lower per cell N content and thus lower absolute per cell N_2_ fixation rates for an equal isotopic enrichment. While our transfer rates are higher than those reported by Krupke et al. [26], they are quite similar to the rates reported by Martinez-Perez et al. [11] from the subtropical N Atlantic for both UCYN-A1 and UCYN-A2 symbioses.

It was surprising that rate processes in the UCYN-A1/haptophyte symbiosis were at times enhanced by the addition of NO_3_^−^ (Fig. 2d, l, Tables S15 and S19), given the lack of direct NO_3_^−^ utilization (Fig. 2h). Phytoplankton and bacterioplankton are known to release dissolved substances, such as dissolved organic N, P, and C [59], B vitamins [60], and compounds that scavenge dissolved iron (e.g., siderophores, [61]), which have the potential to stimulate the fixation of CO_2_ or N_2_ by haptophytes and diazotrophs, respectively, even on these short time scales (<48 h; [62–64]). Vitamin B_12_ is of particular interest, given that haptophytes are suspected to be B_12_ auxotrophs [65]. The stimulating factor cannot be discerned from these experiments, nor whether CO_2_ fixation by the haptophyte or N_2_ fixation by the symbiont is directly stimulated. Further research is needed to identify the mechanism(s) of stimulation. However, experiments where the C fixation rate to N transfer rate ratio is less than the expected cellular C:N of 6.3 [11] demonstrate that an external C source may be required to meet cellular biomass demands (NO3.1, NO3.2, NH4.1; Fig. 4a, c).

In conclusion, this is the first direct evidence that the UCYN-A/haptophyte symbiosis does not assimilate NO_3_^−^, takes up little NH_4_^+^ relative to N demands, and relies on N_2_ fixation as its primary source of N in N-replete waters. These findings add to the growing body of evidence that N_2_ fixation by one of the most widespread and important marine diazotrophs, the UCYN-A/haptophyte symbiosis, is not inhibited by DIN. However, the availability of DIN to the co-existing phytoplankton community may indirectly influence N_2_ fixation and C fixation by the UCYN-A/haptophyte symbiosis. Current ecosystem and biogeochemical models predict little N_2_ fixation in high latitude and temperate coastal regions [66, 67], contrary to recent reports of UCYN-A/haptophyte symbioses (and possibly other active diazotrophs) along with N_2_ fixation in these regions [22, 68–70]. These insights into the biology of the UCYN-A/haptophyte symbioses may enable their inclusion in these models and improve our ability to predict the magnitude and distribution of N_2_ fixation in environments previously considered unimportant with respect to diazotrophy.

## Supplementary information

UCYN-A DIN IMSEJ Revision Supp Clean

Table S13

Table S14

## Data Availability

All data are available in the main text or the supplementary materials.
