# Supplementary material for: Unusual marine cyanobacteria/haptophyte symbiosis relies on N2 fixation even in N-rich environments
Source: ISME J. 2020 Jun 10;14(10):2395–406. doi: 10.1038/s41396-020-0691-6 (PMC7490277; doi:10.1038/s41396-020-0691-6)
Supplement: Supplementary file 1 — UCYN-A DIN IMSEJ Revision Supp Clean [file 41396_2020_691_MOESM1_ESM.docx]

Supplementary Materials

**Title: Unusual marine cyanobacteria/haptophyte symbiosis relies on N_2_**

**fixation even in N-rich environments**

**Authors:** Matthew M. Mills^1*†^, Kendra A. Turk-Kubo^2*†^, Gert L. van Dijken^1^, Britt A. Henke^2^, Katie Harding^2^, Samuel T. Wilson^3^, Kevin R. Arrigo^1^, Jonathan P. Zehr^2^

**Supplementary Materials**

Supplemental Figures S1-S6.

Supplemental Tables S1-S23 (Tables S13-S14 are provided as separate spreadsheets).

Supplemental text.

*Using quantitative PCR to estimate UCYN-A abundance*

*Biomass increase in NH4.1*

**
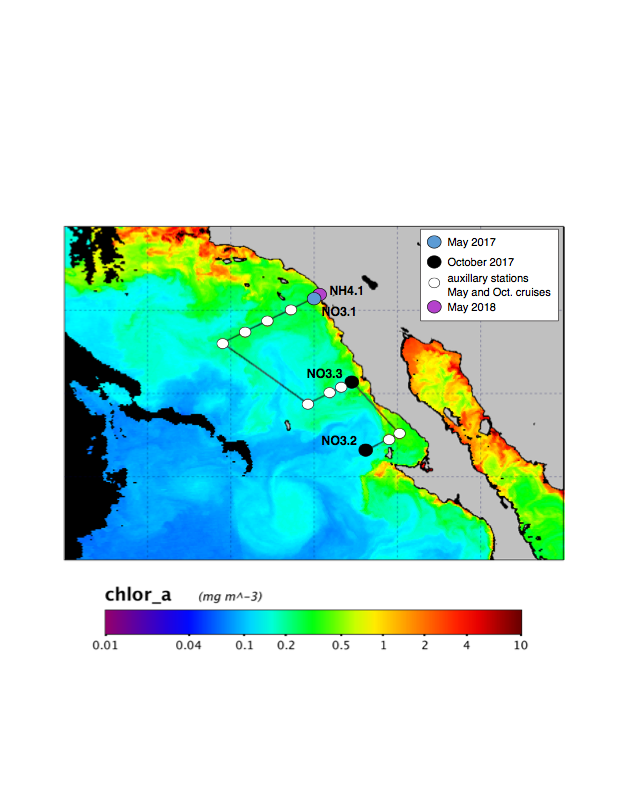
**

**Fig. S1. Sampling locations for NO_3_^-^ and NH_4_^+^-addition experiments.** Stations are overlain on satellite-derived chlorophyll (Chl) *a* data. NO3.1 was conducted on the *R/V Robert Gordon Sproul* in the proximity of the SIO pier in May 2017 (blue dot). NO3.2 and NO3.3 were conducted at stations off the coast of Baja California Sur, Mexico in October 2017 (black dots). NH4.1 was conducted at the SIO pier in May 2018 (purple dot). Chl *a* data is a composite of scenes acquired by two ocean color sensors, MODIS/Aqua and Suomi-NPP/VIIRS, on October 24, 2017.


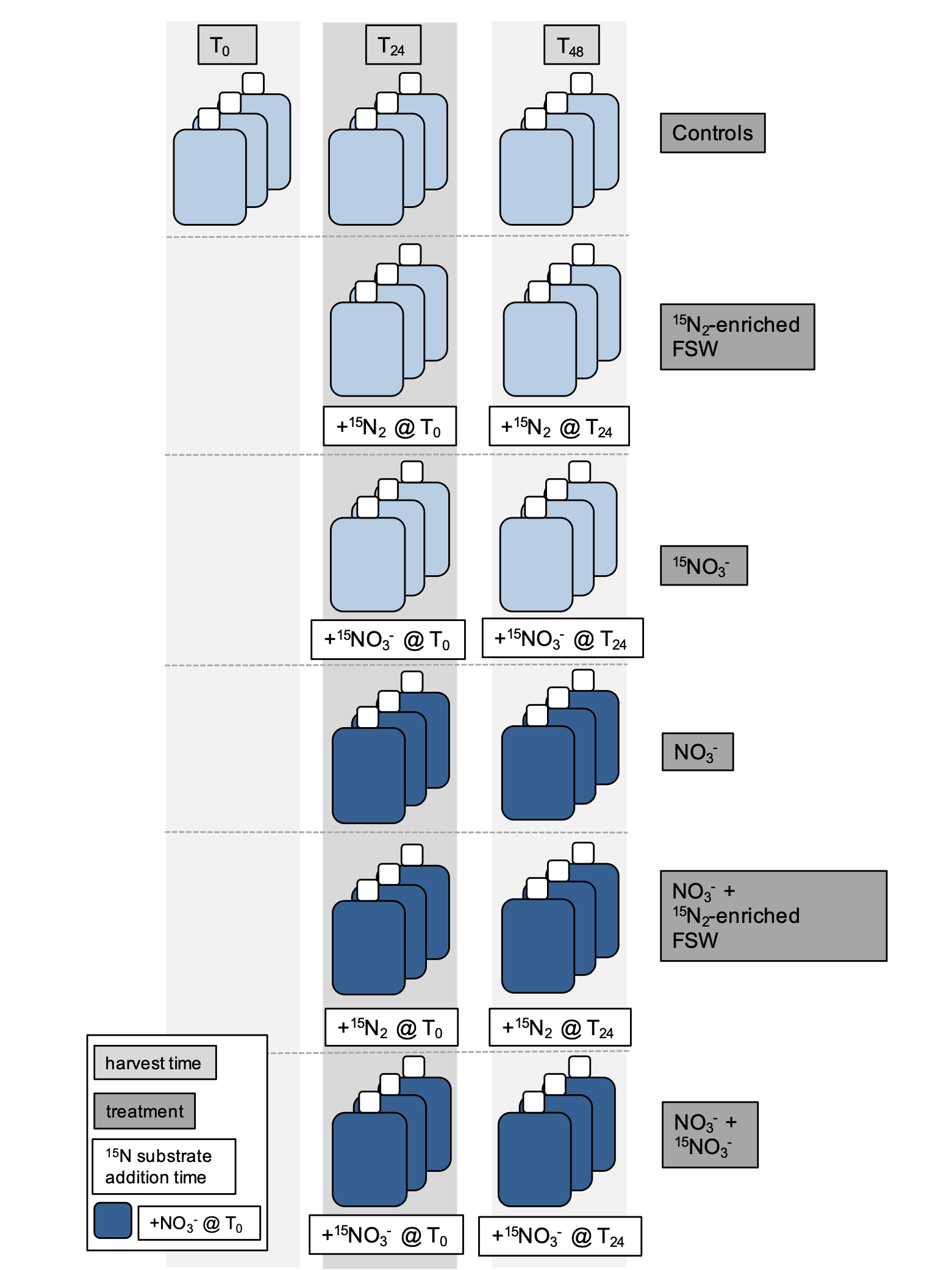


Fig. S2. Experimental design for NO3.1, NO3.2, NO3.3. Treatments are designated in dark grey boxes, time points in light grey boxes, and the timing of ^15^N-substrate additions in white boxes. Control (light blue) and + NO_3_^-^ treatments (dark blue) were in triplicate 4 L bottles incubated at 20% PAR. 2 µM NO_3_^-^ additions were added at T_0_.


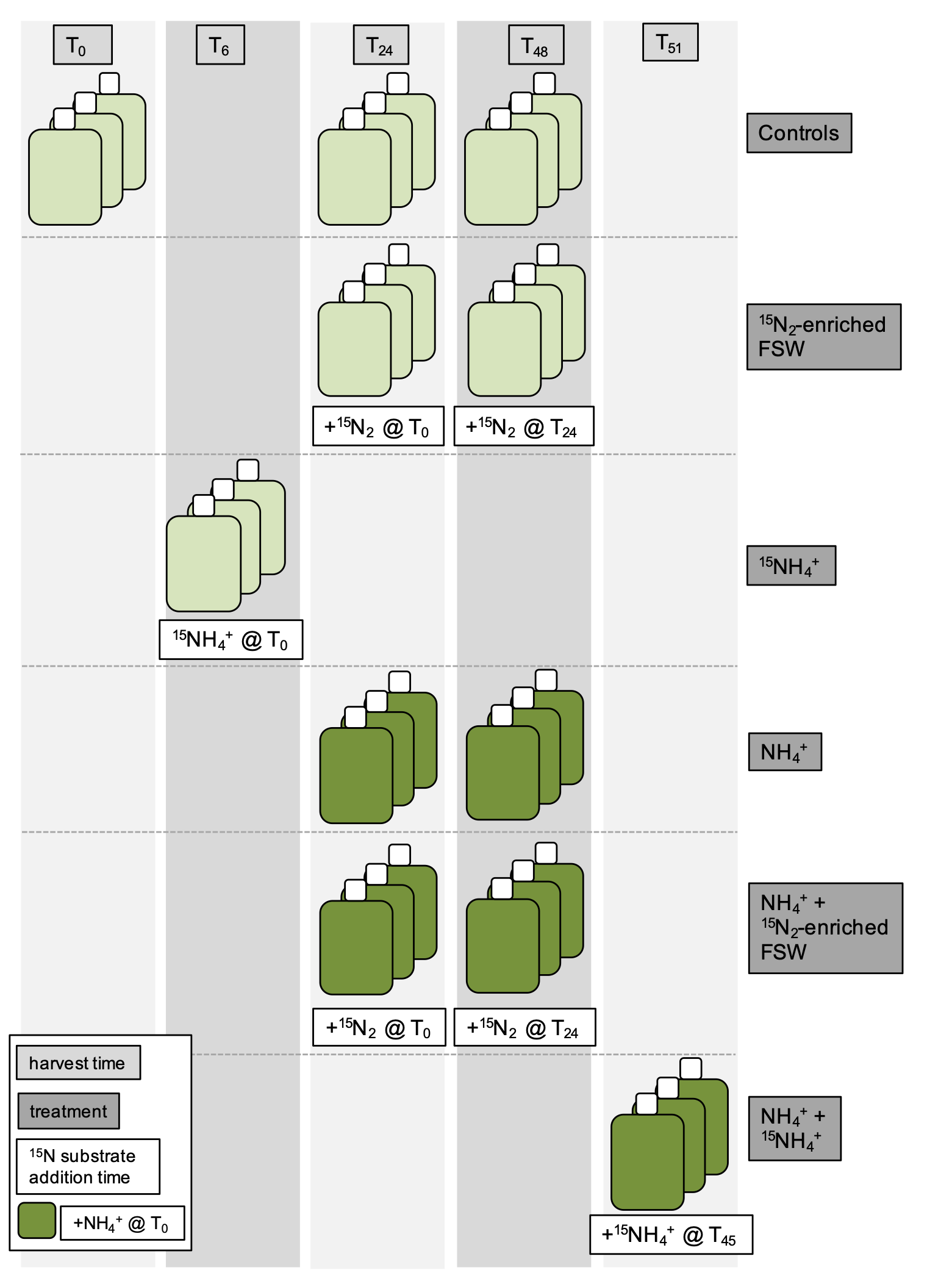


Fig. S3. Experimental design for NH4.1. Treatments are designated in dark grey boxes, time points in light grey boxes, and the timing of ^15^N-substrate additions in white boxes. Control (light green) and + NH_4_^+^ treatments (dark green) were in triplicate 4 L bottles incubated at 20% PAR. 2 µM NH_4_^+^ additions (dark green) were added at T_0_. Note that NH_4_^+^ uptake rates were only measured from T_0_ and T_45_ +NH_4_^+^ waters.

*

*

*

*

*

*

*

*

**Fig. S4.** Bulk particulate (Chl *a*, POC, PON) (A, B) and biological rate processes (CO_2_ fixation, NO_3_^-^ uptake, and N_2_ fixation rates) (C, D) for experiments NO3.2 (A,C) and NO3.3 (B.D). Initial (0-24h) values are denoted with dotted grey lines. ‘*’ indicates treatment means are significantly different (p < 0.05, α = 0.05).

**
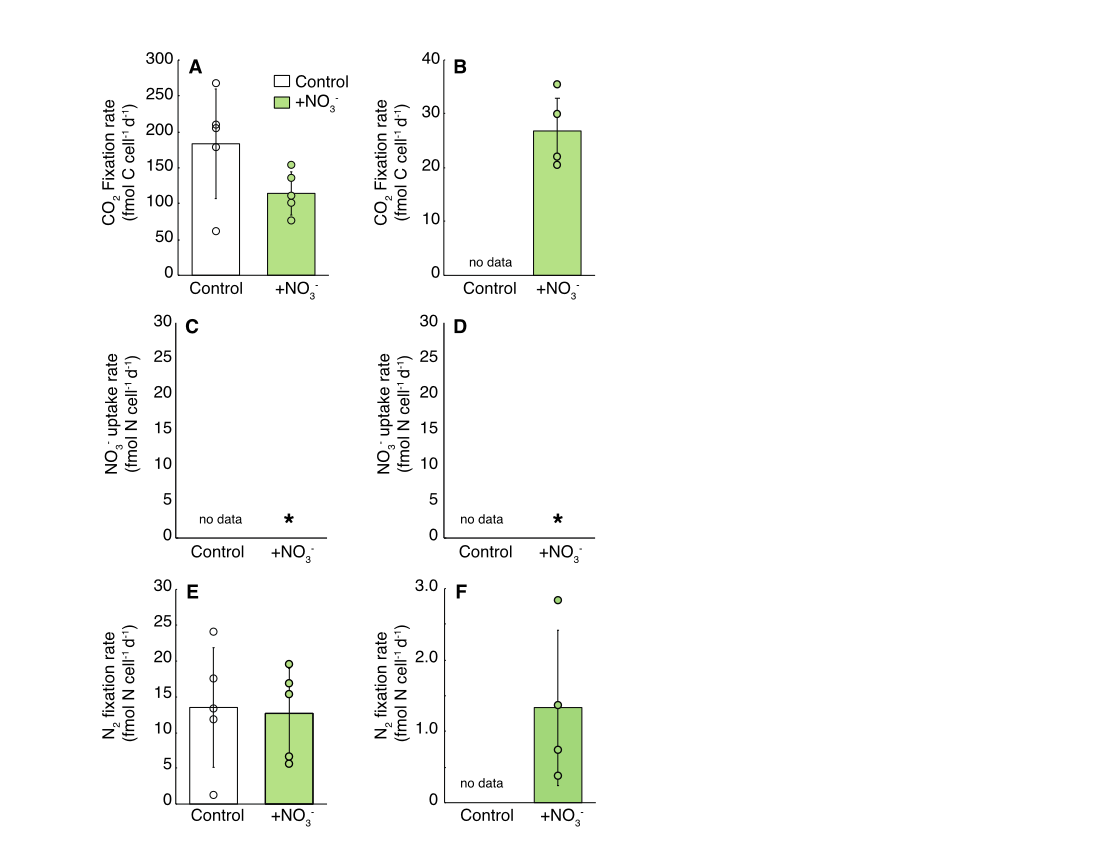
**

BDL

BDL

**Fig. S5.** Cell-specific CO_2_ fixation (A, B), NO_3_^-^ uptake (C, D), and N_2_ fixation (E, F) rates in the NO3.2 and NO3.3 experiments. Measurements from NO3.2 (A,C,E) were made on the UCYN-A2/haptophyte symbiosis. Measurements from NO3.3 (B,D,F) were made on the UCYN-A1/haptophyte symbiosis. In NO3.3 only cells within the +NO_3_^-^ treatment were measured. No NO_3_^-^ uptake was detected (BDL) in any of the UCYN-A/haptophyte symbioses measured for either experiment.

­

Figure S6. ^12^C^14^N images of cells presented in Figures 2 and 3. Panels A-C correspond to cells in Figure 2 A, E, and I respectively. Panels D and E correspond to cells in Figure 3 G and K respectively.


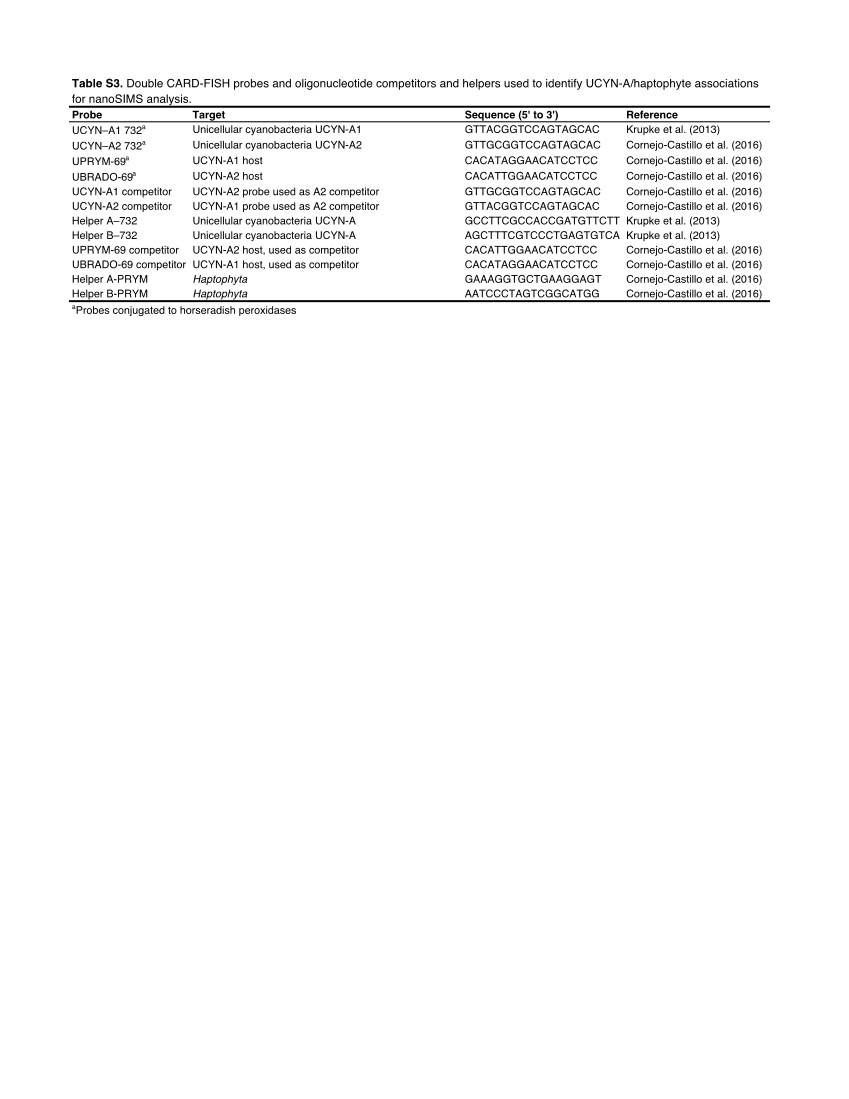


Table S1. Double CARD-FISH probes and oligonucleotide competitors and helpers used to identify UCYN-A/haptophyte symbioses for nanoSIMS analysis.


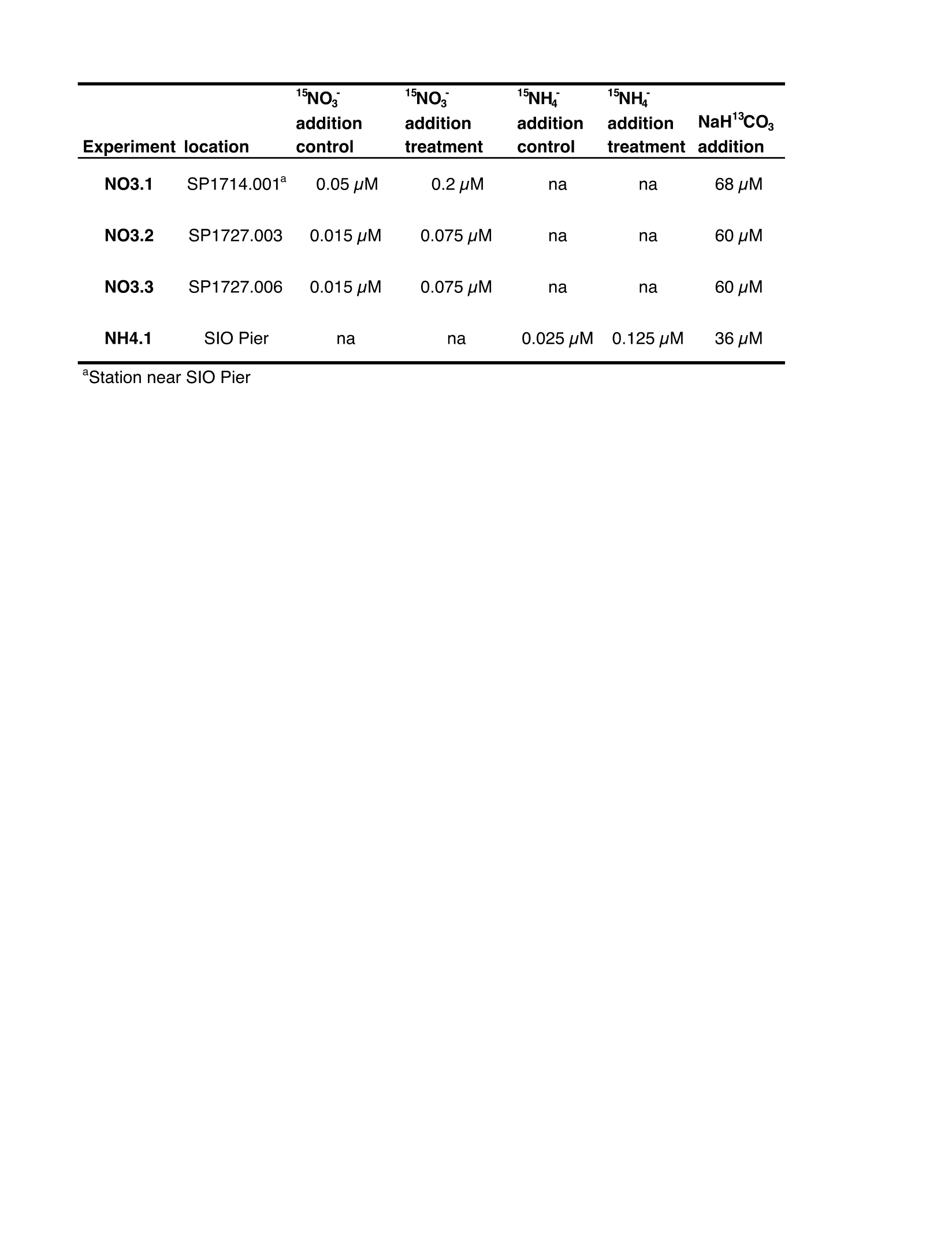


Table S2. Concentrations for labeled substrates added in each experiment. na – not applicable.


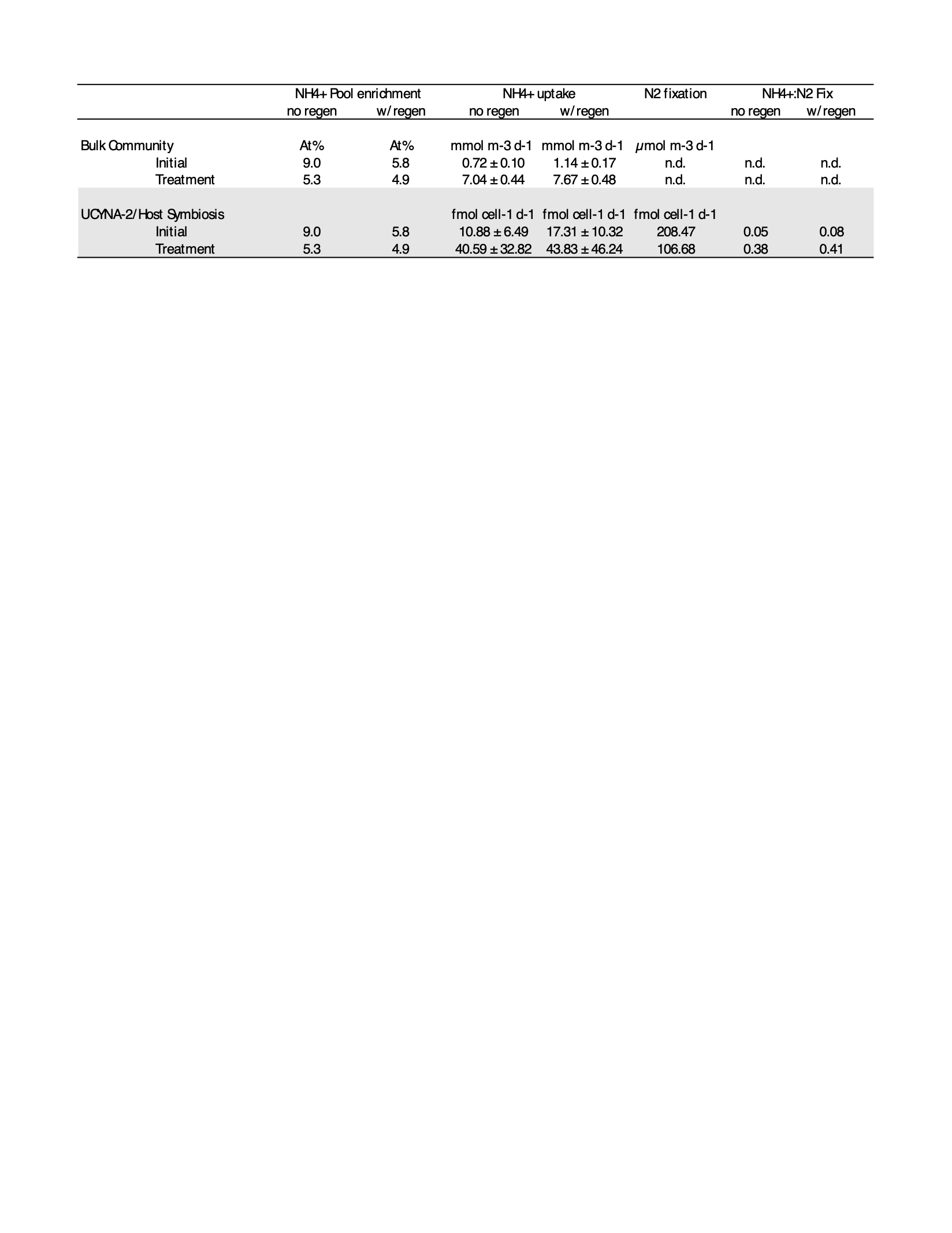


Table S3. Estimates of isotope dilution effects on NH_4_^+^ uptake rates. Estimates of NH_4_^+^ regeneration rates from Bronk and Ward (2005) were used to adjust the enrichment of NH_4_^+^ pool during the uptake rate incubations.

**Table S4.** Summary of bulk rate measurements, limits of detection (LOD), and minimum quantifiable rates (MQR).

Table S5. qPCR-based estimates of UCYN-A abundance in experiments. ud - undetected; dnq - detected below levels of quantitation.

**Table S6.** Single Factor ANOVA comparing responses in bulk Chl *a* concentrations (mg m^-3^) to +NO_3_^-^ treatments for the NO3.1, NO3.2 and NO3.3 experiments and the +NH_4_^+^ treatments for NH4.1 experiment.

**Table S7.** Single Factor ANOVA comparing responses in bulk POC concentrations (mmol m^-3^) to +NO_3_^-^ treatments for the NO3.1, NO3.2 and NO3.3 experiments.

**Table S8.** Single Factor ANOVA comparing responses in bulk PON concentrations (mmol m^-3^) to +NO_3_^-^ treatments for the NO3.1, NO3.2 and NO3.3 experiments and +NH_4_^+^ treatments for the NH4.1 experiment.

**Table S9.** Single Factor ANOVA comparing UCYN-A *nifH*-based abundances to +NO_3_^-^ treatments for the NO3.1 and +NH_4_^+^ treatments for the NH4.1 experiment.

**Table S10.** Single Factor ANOVA comparing bulk community N_2_ fixation rate (nmol m^-3^ d^-1^) responses to +NO_3_^-^ treatments for the NO3.1, NO3.2 and NO3.3 experiments and +NH_4_^+^ treatments for the NH4.1 experiment.

**Table S11.** Single Factor ANOVA comparing bulk community C-fixation rate (mmol m^-3^ d^-1^) responses to +NO_3_^-^ treatments for the NO3.1, NO3.2 and NO3.3 experiments.

**Table S12.** Single Factor ANOVA comparing bulk community NO_3_^-^ uptake responses to +NO_3_^-^ treatments in the NO3.1, NO3.2 and NO3.3 experiments and +NH_4_^+^ uptake responses to +NH_4_^+^ treatments in the NH4.1 experiment.

**Table S13. *See excel file.*** Summary of single cell N_2_ fixation rate measurements, limits of detection (LOD), and minimum quantifiable rates (MQR).

**Table S14. *See excel file.*** Summary of single cell CO_2_ fixation rate measurements, limits of detection (LOD), and minimum quantifiable rates (MQR).

| **Table S15.** Single Factor ANOVA comparing N_2_ fixation rate of the UCYN-A1/haptophyte symbiosis in control and +NO_3_^-^ treatments for NO3.1 experiment. | | | | | | |
| --- | --- | --- | --- | --- | --- | --- |
| SUMMARY |  |  |  |  |  |  |
| *Groups* | *Count* | *Sum* | *Average* | *Variance* |  |  |
| Control N2 fix | 10 | 35.13 | 3.51 | 5.69 |  |  |
| +NO3 N2 Fix | 7 | 48.23 | 6.89 | 1.78 |  |  |
|  |  |  |  |  |  |  |
| ANOVA |  |  |  |  |  |  |
| *Source of Variation* | *SS* | *df* | *MS* | *F* | *P-value* | *F crit* |
| Between Groups | 46.96 | 1 | 46.96 | 11.38 | 0.004 | 4.54 |
| Within Groups | 61.89 | 15 | 4.13 |  |  |  |
|  |  |  |  |  |  |  |
| Total | 108.84 | 16 |  |  |  |  |

| **Table S16.** Single Factor ANOVA comparing N_2_ fixation rate of the UCYN-A2/haptophyte symbiosis in control and +NO_3_^-^ treatments for NO3.2 experiment.  SUMMARY | | | | | | |
| --- | --- | --- | --- | --- | --- | --- |
| *Groups* | *Count* | *Sum* | *Average* | *Variance* |  |  |
| Control N2 fix | 5 | 135.01 | 27.00 | 280.19 |  |  |
| +NO3 N2 Fix | 4 | 198.38 | 49.59 | 769.03 |  |  |
|  |  |  |  |  |  |  |
| ANOVA |  |  |  |  |  |  |
| *Source of Variation* | *SS* | *df* | *MS* | *F* | *P-value* | *F crit* |
| Between Groups | 1134.22 | 1 | 1134.22 | 2.32 | 0.17 | 5.59 |
| Within Groups | 3427.85 | 7 | 489.69 |  |  |  |
|  |  |  |  |  |  |  |
| Total | 4562.07 | 8 |  |  |  |  |

| **Table S17.** Single Factor ANOVA comparing N_2_ fixation rate of the UCYN-A1/haptophyte symbiosis in control and +NH_4_^+^ treatments for NH4.1 experiment. | | | | | | |
| --- | --- | --- | --- | --- | --- | --- |
| SUMMARY |  |  |  |  |  |  |
| *Groups* | *Count* | *Sum* | *Average* | *Variance* |  |  |
| Control N2 fix | 5 | 27.71 | 5.54 | 1.36 |  |  |
| NFR_+NH4 | 6 | 41.99 | 7.00 | 1.82 |  |  |
|  |  |  |  |  |  |  |
| ANOVA |  |  |  |  |  |  |
| *Source of Variation* | *SS* | *df* | *MS* | *F* | *P-value* | *F crit* |
| Between Groups | 5.79 | 1 | 5.79 | 3.59 | 0.09 | 5.12 |
| Within Groups | 14.51 | 9 | 1.61 |  |  |  |
|  |  |  |  |  |  |  |
| Total | 20.30 | 10 |  |  |  |  |

| **Table S18**. Single Factor ANOVA comparing N_2_ fixation rate of the UCYN-A2/haptophyte symbiosis in control and +NH_4_^+^ treatments for NH4.1 experiment. | | | | | | |
| --- | --- | --- | --- | --- | --- | --- |
| SUMMARY |  |  |  |  |  |  |
| *Groups* | *Count* | *Sum* | *Average* | *Variance* |  |  |
| Control_NFR | 4 | 433.25 | 108.31 | 694.80 |  |  |
| +NH4_NFR | 5 | 533.41 | 106.68 | 604.10 |  |  |
|  |  |  |  |  |  |  |
| ANOVA |  |  |  |  |  |  |
| *Source of Variation* | *SS* | *df* | *MS* | *F* | *P-value* | *F crit* |
| Between Groups | 5.91 | 1 | 5.91 | 0.01 | 0.93 | 5.59 |
| Within Groups | 4500.80 | 7 | 642.97 |  |  |  |
|  |  |  |  |  |  |  |
| Total | 4506.71 | 8 |  |  |  |  |

| **Table S19.** Single Factor ANOVA comparing CO_2_ fixation rate of the UCYN-A1/haptophyte symbiosis in control and +NO_3_^-^ treatments for NO3.1 experiment. | | | | | | |
| --- | --- | --- | --- | --- | --- | --- |
| SUMMARY |  |  |  |  |  |  |
| *Groups* | *Count* | *Sum* | *Average* | *Variance* |  |  |
| Control CO2 fix | 10 | 129.67 | 12.97 | 35.25 |  |  |
| +NO3 CO2 Fix | 7 | 200.09 | 28.58 | 57.42 |  |  |
|  |  |  |  |  |  |  |
| ANOVA |  |  |  |  |  |  |
| *Source of Variation* | *SS* | *df* | *MS* | *F* | *P-value* | *F crit* |
| Between Groups | 1004.39 | 1 | 1004.39 | 22.77 | 0.0002 | 4.54 |
| Within Groups | 661.78 | 15 | 44.12 |  |  |  |
|  |  |  |  |  |  |  |
| Total | 1666.17 | 16 |  |  |  |  |

| **Table S20.** Single Factor ANOVA comparing CO_2_ fixation rate of the UCYN-A2/haptophyte symbiosis in control and +NO_3_^-^ treatments for NO3.2 experiment. | | | | | | |
| --- | --- | --- | --- | --- | --- | --- |
| SUMMARY |  |  |  |  |  |  |
| *Groups* | *Count* | *Sum* | *Average* | *Variance* |  |  |
| Control CO2 fix | 7 | 420.78 | 60.11 | 638.55 |  |  |
| +NO3 CO2 Fix | 7 | 533.71 | 76.24 | 1160.01 |  |  |
|  |  |  |  |  |  |  |
| ANOVA |  |  |  |  |  |  |
| *Source of Variation* | *SS* | *df* | *MS* | *F* | *P-value* | *F crit* |
| Between Groups | 910.97 | 1 | 910.97 | 1.01 | 0.33 | 4.75 |
| Within Groups | 10791.32 | 12 | 899.28 |  |  |  |
|  |  |  |  |  |  |  |
| Total | 11702.29 | 13 |  |  |  |  |

| **Table S21.** Single Factor ANOVA comparing CO_2_ fixation rate of the UCYN-A1/haptophyte symbiosis in control and +NH_4_^+^ treatments for NH4.1 experiment. | | | | | | |
| --- | --- | --- | --- | --- | --- | --- |
| SUMMARY |  |  |  |  |  |  |
| *Groups* | *Count* | *Sum* | *Average* | *Variance* |  |  |
| Control CO2 fix | 5 | 70.09 | 14.02 | 115.62 |  |  |
| +NH4 CO2 Fix | 6 | 25.64 | 4.27 | 17.47 |  |  |
|  |  |  |  |  |  |  |
| ANOVA |  |  |  |  |  |  |
| *Source of Variation* | *SS* | *df* | *MS* | *F* | *P-value* | *F crit* |
| Between Groups | 258.93 | 1 | 258.93 | 4.24 | 0.07 | 5.12 |
| Within Groups | 549.84 | 9 | 61.09 |  |  |  |
|  |  |  |  |  |  |  |
| Total | 808.77 | 10 |  |  |  |  |

| **Table S22.** Single Factor ANOVA comparing CO_2_ fixation rate of the UCYN-A2/haptophyte symbiosis in control and +NH_4_^+^ treatments for NH4.1 experiment. | | | | | | |
| --- | --- | --- | --- | --- | --- | --- |
| SUMMARY |  |  |  |  |  |  |
| *Groups* | *Count* | *Sum* | *Average* | *Variance* |  |  |
| Control_CFR | 4 | 1409.85 | 352.46 | 8460.59 |  |  |
| +NH4_CFR | 5 | 1989.62 | 397.92 | 8420.74 |  |  |
|  |  |  |  |  |  |  |
| ANOVA |  |  |  |  |  |  |
| *Source of Variation* | *SS* | *df* | *MS* | *F* | *P-value* | *F crit* |
| Between Groups | 4592.93 | 1 | 4592.93 | 0.54 | 0.48 | 5.59 |
| Within Groups | 59064.71 | 7 | 8437.82 |  |  |  |
|  |  |  |  |  |  |  |
| Total | 63657.64 | 8 |  |  |  |  |

**Table S23.** qPCR-based estimates of N_2_-fixer abundance at T0. ud - undetected; dnq - detected below levels of quantitation.

Supplemental Text

Supplemental Methods

*Using quantitative PCR to estimate UCYN-A abundances*

DNA samples were immediately filtered onto Sterivex (MilliporeSigma, Burlington, MA) using gentle peristaltic pumping, then flash frozen in liquid N_2_, and stored at -80^o^C until extraction. DNA was extracted using a bead-beating protocol ^34^ with modifications to remove the filter from the sterivex cartridge and automated the on-column steps of the protocol using a QIACube (Qiagen, Germantown, MD). DNA quality was assessed using an Agilent 2100 Bioanalyzer (Santa Clara, CA) and concentrations were determined using a Picogreen assay (ThermoFisher Scientific, Waltham, MA).

We quantified gene-based abundances of UCYN-A1 and UCYN-A2/A3 using Taqman® qPCR assays ^6,35^. The qPCR assay designed by Thompson et al., (2015) amplifies UCYN-A2, UCYN-A3, and UCYN-A4. At SIO, the UCYN-A assemblage is dominated by UCYN-A2, with UCYN-A4 reported at low relative abundance. UCYN-A3 is not detected (Cornejo-Castillo et al., 2018). Therefore, *nifH*-based estimates of UCYN-A2 abundance reported here may also include UCYN-A4. Details of qPCR standard generation, plate design, thermocycling parameters, inhibition tests, determination of the limit of detection (LOD) and quantification (LOQ), as well as abundance calculations are described in detail in ^36^. The LOD and LOQ for all assays were 13 and 100 *nifH* copies L^-1^, respectively. It should be noted that genome copy numbers in the UCYN-A haptophyte symbiosis are unknown, therefore, qPCR-based abundances must be interpreted with caution and cannot be translated into cell numbers.

Supplemental Discussion

*Biomass increase in NH4.1.*

In the NH4.1 treated samples the PON and POC increased ~ 10 and 40 µmol L^-1^ respectively, a much greater response than expected from the 2 µmol L^-1^ NH_4_^+^ addition. We hypothesize that the excess growth in NH4.1 was fueled by luxury N uptake by the active bloom of the dinoflagellate *Lingulodinium polyedra* and/or consumption of DON. Concentrations of NO_3_^-^ on April 30th (10 days prior to the experiment) were 6 µmol L^-1^. It is likely that a portion of this uptake was stored, and then fueled the excess POC and PON increase beyond the treatment N addition. Additionally, dinoflagellate uptake of DON is well documented (*60*), and may have supplemented any further excess particulate growth.
